# Incidence and Predictors of In-Hospital Mortality Among Diabetics Undergoing Transcatheter Aortic Valve Replacement

**DOI:** 10.7759/cureus.16056

**Published:** 2021-06-30

**Authors:** Jannat Malik, Jeet R Patel, Hajara Joundi, Kristal N Pereira, Amanpreet K Pannu, Goher Haneef, Khadija T Kubra, Keerthika Mathialagan, Temitope Ajibawo

**Affiliations:** 1 Family Medicine, National University of Medical Sciences, Rawalpindi, PAK; 2 Pediatrics, Byramjee Jeejeebhoy Medical College, Ahmedabad, IND; 3 Internal Medicine, University Cadi Ayyad, Marrakech, MAR; 4 Internal Medicine, Terna Medical College, Mumbai, IND; 5 Internal Medicine, Sri Guru Ram Das University of Health Sciences, Amritsar, IND; 6 Internal Medicine, University of Health Sciences, Lahore, PAK; 7 Emergency Medicine, University of Cincinnati Medical Center, Cincinnati, USA; 8 Internal Medicine, Bangladesh Medical College and Hospital, Dhaka, BGD; 9 Psychiatry, Sree Balaji Medical College and Hospital, Chennai, IND; 10 Internal Medicine, Brookdale University Hospital Medical Center, New York City, USA

**Keywords:** new generation tavr, trans-catheter aortic valve replacement, critical aortic stenosis, mortality, diabetes mellitis

## Abstract

Objectives

The main goals of this study are to delineate the differences in demographics, comorbidities and hospital outcomes between diabetic and non-diabetic aortic stenosis (AS) patients, and next is to evaluate the predictors of in-hospital mortality in AS patients undergoing transcatheter aortic valve replacement (TAVR).

Methods

We conducted an observational cross-sectional study using the nationwide inpatient sample (NIS) and included 33,325 adult patients with a primary discharge diagnosis of AS who underwent TAVR during the hospitalization. This sample was further grouped by comorbid diabetic which include non-diabetics (N = 23,585) versus diabetic patients (N = 9,740). Among the hospital outcomes we included the length of stay (LOS) and total cost during hospitalization, and the all-cause in-hospital mortality. We used an independent logistic regression model adjusted for demographic confounders to measure the adjusted odds ratio (aOR) of association of comorbid medical conditions and in-hospital mortality risk in non-diabetic and diabetic groups.

Results

The most prevalent medical comorbidities among inpatients with diabetes were hypertension (85.1%), followed by renal failure (38.0%), chronic lung disease (37.1%), obesity (21.3%), and these values were significantly higher compared with the non-diabetic group. The in-hospitality mortality was higher among the non-diabetic group (4.7%) compared to the diabetic group (2.8%). There was no significant difference in mean length of stay and mean total cost between the diabetic and non-diabetic groups. In diabetic AS inpatients, stroke (aOR: 4.58, 95%CI: 2.23-9.42) and fluid/electrolyte disorders (aOR: 4.25, 95%CI: 3.29-5.48) had a statistically significant association with mortality risk when compared to the non-diabetic group. Among the non-diabetic AS inpatients, fluid/electrolyte disorders had the highest mortality risk (aOR: 2.48, 95% CI 2.17-2.83) followed by coagulopathy (aOR: 2.03; CI: 1.77-2.32), congestive heart failure (aOR: 1.67; CI: 1.40-1.98), and renal failure (aOR: 1.62; CI: 1.41-1.86). Meanwhile, hypertension and obesity had a statistically non-significant and negative association with in-hospital mortality in diabetic and non-diabetic groups.

Conclusions

Diabetic AS inpatients following TAVR had a higher mortality risk with comorbid fluid/electrolyte disorders and stroke. In-hospital mortality following TAVR was lower among the diabetics compared to non-diabetics, and it underscores diabetes as a surgical risk factor in patients with AS. So, TAVR may be the preferred approach for diabetic patients with AS along with cardiovascular risk factor modification, strict glycemic control and timely renal function follow-up.

## Introduction

Aortic stenosis (AS) is one of the most prevailing valvulopathies in the world and about 61% of all valvular deaths are due to aortic valve diseases [1]. The prevalence of AS increases with age, 2.5% in those between 75-76 years of age to 8.1% in those over 85 [2]. Female to male sex ratio increases with age, but five-year mortality rates are lower in females [3]. The prevalence of severe AS is significantly higher in whites when compared to blacks [4].

Both, AS and diabetes are progressive diseases that carry significant morbidity and mortality. Diabetes is a well-established risk factor for developing atherosclerosis and cardiovascular disease [5]. Recent studies have shown that the prevalence of diabetes is higher in patients with AS than in the general population, and ranges between 11.4% to 34% [6,7]. Comorbid diabetes alters the outcome of AS, wherein it increases the rate of progression of the disease [8]. Inflammation and lipid accumulation contribute to the progressive calcification process of AS on either a defected or normal aging valve. Valvular heart diseases share similar risk factors with atherosclerotic diseases, including diabetes and hypertension [9]. AS in turn leads to ventricular hypertrophic remodeling and diastolic heart failure. Based on the European system for cardiac operative risk evaluation score, diabetes is considered a marker of poor prognosis in patients with AS [10]. Mild asymptomatic patients are treated conservatively with medical management. Meanwhile, for symptomatic patients and those with severe AS, aortic valve replacement is the only definitive treatment [11]. About 80% of patients with severe asymptomatic AS will need aortic valve replacement or they have higher chances to die within four years [12].

Several comorbid conditions are associated with increased morbidity and mortality after transcatheter aortic valve replacement (TAVR) [13]. Following TAVR, diabetic patients seem to have similar peri-procedural and mid-term outcomes compared with patients without diabetes. Diabetic patients have an increased risk of vascular and bleeding complications up to 30-day post-TAVR period, acute kidney injury (AKI), and one‐year mortality [14-16]. The main goals of this study are to delineate the differences in demographics, comorbidities, and hospital outcomes between diabetic and non-diabetic patients, and next is to evaluate the predictors of in-hospital mortality in diabetic and non-diabetic AS patients undergoing TAVR.

## Materials and methods

Study sample

We conducted an observational cross-sectional study using the nationwide inpatient sample (NIS) from 4,411 hospitals across 44 states in the United States. As, the NIS is a de-identified publicly available data with the protection of patient’s health information, so we were not required to take institutional review board permission for this study [17].

We included 33,325 adult patients with a primary discharge diagnosis of AS who underwent TAVR during the hospitalization. The diagnosis of AS was identified using the International Classification of Diseases, ninth revision (ICD-9) diagnosis codes of 395.0, 395.2, 396.0, 396.2, 424.1 or 746.3, and the procedure of TAVR identified using ICD-9 procedure codes of 350.5 or 350.6. This sample was further grouped by a comorbid diagnosis of diabetes which includes non-diabetics (N = 23,585) versus diabetic patients (N = 9,740).

Variables

Demographic variables included were age, sex, and race [18]. Comorbid medical conditions included in the study are hypertension, obesity, congestive heart failure (CHF), chronic lung disease (CLD), coagulopathy, stroke, renal failure, and fluid/electrolyte disorders, and peripheral vascular disorders (PVD). Among the hospital outcomes, we included the length of stay (LOS) and total cost during hospitalization, and the all-cause in-hospital mortality. LOS in the NIS is calculated by the number of nights the patient was hospitalized for AS and the total cost during hospitalization excludes professional fees and other non-covered charges [18]. 

Statistical analysis

We compared the distributions of demographic characteristics and comorbid medical conditions, and hospital outcomes between non-diabetic versus diabetic groups by performing descriptive statistics and Pearson’s chi-square test. Next, we measured the differences in continuous variables, that is, age, LOS, and total cost between the non-diabetic versus diabetic groups using the independent sample T-test. We used an independent logistic regression model adjusted for demographic confounders (of age, sex, and race) to measure the adjusted odds ratio (aOR) of association of comorbid medical conditions and in-hospital mortality risk in non-diabetic and diabetic groups. All analyses were conducted using the Statistical Package for the Social Sciences (SPSS), version 26.0 (IBM Corp., Armonk, NY) and statistical significance was set at a two-sided P-value <0.001. 

## Results

Our study population included 33,325 adult inpatients with AS that underwent TAVR. Among the inpatients, 29.2% had diabetes, the majority of whom were males (54.1%) and Caucasians (86.2%).

The most prevalent medical comorbidities among inpatients with diabetes were hypertension (85.1%), followed by renal failure (38.0%), chronic lung disease (37.1%), obesity (21.3%), and these values were significantly higher compared with the non-diabetic group. Meanwhile, fluid/electrolyte disorders (27.1%), coagulopathy (23.9%), stroke (1.3%), and congestive heart failure (12.2%) were more prevalent among the non-diabetic group.

The in-hospitality mortality was higher among the non-diabetic group (4.7%) compared to the diabetic group (2.8%). There was no significant difference in mean LOS and mean total cost between the diabetic and non-diabetic groups as shown in Table 1.

**Table 1:** Difference in aortic stenosis in patients undergoing transcatheter aortic valve replacement. SD: standard deviation; $: United States dollars.

Variable	Non-diabetic	Diabetic	P-value
Mean age (SD), in years	82.1 (7.9)	79.7 (7.9)	<0.001
Sex, in %			
Male	51.1	54.1	<0.001
Female	48.9	45.9	
Race, in %			
White	89.8	86.2	<0.001
Black	3.4	4.6	
Hispanic	2.4	4.1	
Others	4.4	5.1	
Comorbid medical conditions, in %			
Hypertension	78.3	85.1	<0.001
Obesity	11.3	21.3	<0.001
Congestive heart failure	12.2	10.6	<0.001
Chronic lung disease	32.5	37.1	<0.001
Coagulopathy	23.9	22.3	0.02
Stroke	1.3	0.8	<0.001
Renal failure	34.3	38.0	<0.001
Fluid/electrolyte disorders	27.1	24.5	<0.001
Peripheral vascular disorders	29.7	29.6	0.780
Hospital outcomes			
In-hospital mortality, in %	4.7	2.8	<0.001
Mean length of stay (SD), in days	7.8 (6.8)	7.5 (6.4)	0.06
Mean total cost (SD), in $	214169 (124781)	211345 (119197)	0.06

In diabetic AS inpatients, stroke (aOR: 4.58, 95%CI: 2.23-9.42) and fluid/electrolyte disorders (aOR: 4.25, 95%CI: 3.29-5.48) had a statistically significant association with mortality risk. Among the non-diabetic AS inpatients, fluid/electrolyte disorders had the highest mortality risk (aOR: 2.48, 95% CI 2.17-2.83) followed by coagulopathy (aOR: 2.03; CI: 1.77-2.32), CHF (aOR: 1.67; CI: 1.40-1.98), and renal failure (aOR: 1.62; CI: 1.41-1.86). Meanwhile, hypertension and obesity had a statistically non-significant and negative association with in-hospital mortality in diabetic and non-diabetic groups as shown in Table 2.

**Table 2:** In-hospital mortality risk predictors in aortic stenosis inpatients undergoing transcatheter aortic valve replacement. aOR: adjusted odds ratio.

Comorbidity	Non-diabetic			Diabetic		
	aOR	95% confidence interval	P-value	aOR	95% confidence interval	P-value
Hypertension	0.33	0.29-0.38	<0.001	0.94	0.65-1.36	0.733
Obesity	0.72	0.55-0.93	0.011	0.97	0.71-1.33	0.841
Congestive heart failure	1.67	1.40-1.98	<0.001	1.34	0.94-1.91	0.104
Chronic lung disease	1.13	0.98-1.29	0.101	0.79	0.61-1.03	0.083
Coagulopathy	2.03	1.77-2.32	<0.001	1.25	0.95-1.64	0.114
Stroke	1.21	0.75-1.96	0.429	4.58	2.23-9.42	<0.001
Renal failure	1.62	1.41-1.86	<0.001	1.39	1.08-1.81	0.011
Fluid/electrolyte disorders	2.48	2.17-2.83	<0.001	4.25	3.29-5.48	<0.001
Peripheral vascular disorders	1.21	1.05-1.39	0.008	1.42	1.10-1.84	0.007

## Discussion

Although AS patients undergoing TAVR were elderly, the mean age among inpatients undergoing TAVR was lower among the diabetic group than the non-diabetic group in our study. The aortic valve undergoes remodeling with increasing age. Diabetes contributes to further inflammation and calcium deposits, increasing the chances of a severe AS and an earlier presentation [19]. 

Among the diabetic cohort in our study, the majority were males and whites. Based on a study by Patel et al., the prevalence of AS was low in blacks compared to whites, and the white race was an independent risk factor for severe AS [4]. Past studies revealed that although underrepresented ethnic groups had a high prevalence of risk factors, they had a lower incidence of AS. However, blacks with severe AS had higher morbidity and mortality than whites [20]. 

In our study, hypertension was a significant comorbid factor among inpatients with diabetes compared to the non-diabetic group. Diabetes promotes atherosclerosis by damaging and hardening the arteries leading to high blood pressure [21]. We also found renal failure to be more prevalent among diabetic patients undergoing TAVR. The higher prevalence of renal failure in the diabetic population could be attributed to a higher risk of acute kidney injury among diabetic patients at baseline [15]. Furthermore, multiple hypotensive episodes following the TAVR procedure could also lead to AKI [15]. 

While renal failure was more prevalent among the diabetic group, the in-hospital mortality risk from renal failure was higher in the non-diabetic group. Past studies have revealed increased mortality rates following TAVR with even minimal reduction in renal function, and diabetes is one among many factors contributing to reduced renal function [22]. Fluid and electrolyte disorders were associated with higher mortality risk among the diabetic group in our study. Electrolyte abnormalities are common in diabetic patients, especially with decompensated diabetes, and are associated with increased morbidity and mortality [23]. Fluid disturbances compounded with poor renal function among diabetic patients could lead to increased mortality risk following TAVR.

Even though the prevalence of stroke among diabetic patients was 0.8%, the mortality risk associated with stroke in the diabetic group was significantly higher compared to the non-diabetic group. Diabetes can cause vascular endothelial dysfunction, increased early-age arterial stiffness, systemic inflammation, and thickening of the basal capillary membrane, contributing to stroke and increased mortality rates eventually [24]. Diabetes also promotes cerebral atherogenesis and exacerbates other risk factors like hypertension, heart disease, and hyperlipidemia. All these lead to an increased risk of stroke among diabetic patients.

Thrombotic events following TAVR are a significant issue. In our study, coagulopathy had an increased association with mortality. The pathogenesis behind thrombosis following TAVR is multifactorial. Flow disturbances, prothrombotic metallic frame, and a prothrombotic tendency could contribute towards thrombosis [25]. Another significant comorbidity associated with increased mortality was congestive heart failure. The finding is consistent with a prior single-center study of 572 patients who underwent TAVR, where patients readmitted for CHF following TAVR had an increased mortality risk [26].

Interestingly, our study found that AS patients undergoing TAVR who had diabetes had lower in-hospital mortality rate. Although the negative impact of diabetes on the prognosis of cardiovascular disease and interventional procedures is well known, past literature on diabetes influence on TAVR outcomes has been conflicting. A previous study by Berkovitch et al. found no statistically significant difference in both short-term and long-term mortality rates between the diabetic and non-diabetic groups [14]. These observations contrast with findings in a study by Mina et al. where diabetes was associated with significantly higher one-year mortality but not early mortality risk [15]. These studies could not establish a causal relationship between diabetes and increased mortality rates.

Our study had some limitations. We conducted an observational cross-sectional study, and data were collected only at a given point of time, providing a snapshot of the relationship between the risk factor and disease outcome and all-cause in-hospital mortality. Hence it could not measure the exact cause and effect relationship. We used a NIS, and it lacked patient-level clinical information. Inpatients were based on ICD-9 codes, so there may be underreporting of comorbidities. However, the main strength of our study was that we used a nationally representative database with a large sample size that increased the power of the study with minimal reporting bias.

## Conclusions

Diabetic AS inpatients following TAVR had a higher mortality risk with comorbid fluid/electrolyte disorders and stroke. In-hospital mortality following TAVR was lower among the diabetics compared to non-diabetics, and it underscores diabetes as a surgical risk factor in patients with AS. So, TAVR may be the preferred approach for diabetic patients with AS along with cardiovascular risk factor modification, strict glycemic control and timely renal function follow-up.

## References

[1] (2021). Valvular heart disease. https://www.cdc.gov/heartdisease/valvular_disease.htm.

[2] Lindroos M, Kupari M, Heikkilä J, Tilvis R (1993). Prevalence of aortic valve abnormalities in the elderly: an echocardiographic study of a random population sample. J Am Coll Cardiol.

[3] Toyofuku M, Taniguchi T, Morimoto T (2017). Sex differences in severe aortic stenosis - clinical presentation and mortality. Circ J.

[4] Patel DK, Green KD, Fudim M, Harrell FE, Wang TJ, Robbins MA (2014). Racial differences in the prevalence of severe aortic stenosis. J Am Heart Assoc.

[5] Martín-Timón I, Sevillano-Collantes C, Segura-Galindo A, Del Cañizo-Gómez FJ (2014). Type 2 diabetes and cardiovascular disease: have all risk factors the same strength?. World J Diabetes.

[6] Taniguchi T, Morimoto T, Shiomi H (2015). Initial surgical versus conservative strategies in patients with asymptomatic severe aortic stenosis. J Am Coll Cardiol.

[7] Reardon MJ, Van Mieghem NM, Popma JJ (2017). Surgical or transcatheter aortic-valve replacement in intermediate-risk patients. N Engl J Med.

[8] Banovic M, Athithan L, McCann GP (2019). Aortic stenosis and diabetes mellitus: an ominous combination. Diab Vasc Dis Res.

[9] Stewart BF, Siscovick D, Lind BK (1997). Clinical factors associated with calcific aortic valve disease. J Am Coll Cardiol.

[10] Deutscher S, Rockette HE, Krishnaswami V (1984). Diabetes and hypercholesterolemia among patients with calcific aortic stenosis. J Chronic Dis.

[11] Marquis-Gravel G, Redfors B, Leon MB, Généreux P (2016). Medical treatment of aortic stenosis. Circulation.

[12] Rosenhek R, Binder T, Porenta G (2000). Predictors of outcome in severe, asymptomatic aortic stenosis. N Engl J Med.

[13] Hermiller JB Jr, Yakubov SJ, Reardon MJ (2016). Predicting early and late mortality after transcatheter aortic valve replacement. J Am Coll Cardiol.

[14] Berkovitch A, Segev A, Barbash I (2015). Clinical impact of diabetes mellitus in patients undergoing transcatheter aortic valve replacement. Cardiovasc Diabetol.

[15] Mina GS, Gill P, Soliman D, Reddy P, Dominic P (2017). Diabetes mellitus is associated with increased acute kidney injury and 1-year mortality after transcatheter aortic valve replacement: a meta-analysis. Clin Cardiol.

[16] Goel R, Power D, Tchetche D (2019). Impact of diabetes mellitus on short term vascular complications after TAVR: results from the BRAVO-3 randomized trial. Int J Cardiol.

[17] (2021). Overview of the National (Nationwide) Inpatient Sample. https://hcup-us.ahrq.gov/nisoverview.jsp.

[18] (2021). NIS description of data elements. https://hcup-us.ahrq.gov/db/nation/nis/nisdde.jsp.

[19] Pradhan AD, Manson JE, Rifai N, Buring JE, Ridker PM (2001). C-reactive protein, interleukin 6, and risk of developing type 2 diabetes mellitus. JAMA.

[20] Wilson JB, Jackson LR 2nd, Ugowe FE, Jones T, Yankey GSA Jr, Marts C, Thomas KL (2020). Racial and ethnic differences in treatment and outcomes of severe aortic stenosis: a review. JACC Cardiovasc Interv.

[21] Kozakova M, Palombo C (2016). Diabetes mellitus, arterial wall, and cardiovascular risk assessment. Int J Environ Res Public Health.

[22] Najjar M, Salna M, George I (2015). Acute kidney injury after aortic valve replacement: incidence, risk factors and outcomes. Expert Rev Cardiovasc Ther.

[23] Liamis G, Liberopoulos E, Barkas F, Elisaf M (2014). Diabetes mellitus and electrolyte disorders. World J Clin Cases.

[24] Chen R, Ovbiagele B, Feng W (2016). Diabetes and stroke: epidemiology, pathophysiology, pharmaceuticals and outcomes. Am J Med Sci.

[25] Ranasinghe MP, Peter K, McFadyen JD (2019). Thromboembolic and bleeding complications in transcatheter aortic valve implantation: insights on mechanisms, prophylaxis and therapy. J Clin Med.

[26] Durand E, Doutriaux M, Bettinger N (2017). Incidence, prognostic impact, and predictive factors of readmission for heart failure after transcatheter aortic valve replacement. JACC Cardiovasc Interv.

